# Endodontic Treatment in Artificial Deciduous Teeth through Manual and Mechanical Instrumentation: A Pilot Study

**DOI:** 10.5005/jp-journals-10005-1581

**Published:** 2019

**Authors:** Fernanda Hecksher, Bruno Vidigal, Patricia Coelho, Diassanam Otoni, Christiano Alvarenga, Eduardo Nunes

**Affiliations:** 1–3,6 Department of Dentistry, PUC-Minas, Minas Gerais, Brazil; 2 Department of Dentistry, Newton Paiva, Minas Gerais, Brazil; 3 Department of Dentistry, Fainor, Minas Gerais, Brazil; 1,4,5 Department of Dentistry, São Leopoldo Mandic, Minas Gerais, Brazil

**Keywords:** Deciduous teeth, Endodontics, Instrumentation, Pediatric dentistry

## Abstract

“*In vitro*” studies seek to simulate actual biological conditions in the laboratory providing principles to be studied later. To facilitate the development of new techniques of root instrumentation in primary teeth and to ensure the integrity and function of the element, this study aimed to demonstrate the technological advances in endodontics through endodontic treatment performed on artificial primary teeth using a rotary instrumentation system and reciprocation. Instrumentation of the root canal was performed *via* a manual, rotary, and Reciproc system. Results showed that current systems can facilitate endodontic treatment in one session. These procedures become increasingly easy with the help of technological advances in dentistry.

**How to cite this article:**

Hecksher F, Vidigal B, *et al.* Endodontic Treatment in Artificial Deciduous Teeth through Manual and Mechanical Instrumentation: A Pilot Study. Int J Clin Pediatr Dent 2019;12(1):15–17.

## INTRODUCTION

The dental pulp of deciduous teeth can become involved much earlier than permanent teeth in the advance of carious lesions. In addition, exposure of the pulp can also occur much more often during cavity preparation due to the thickness of the enamel and dentin, which are very often thinner in deciduous teeth. It is also noteworthy that traumatic injuries, especially in anterior teeth, occur frequently, contributing to a public health problem.^1,2^

The main goal of dentistry is to maintain the integrity and function of the primary dentition to its physiological exfoliation. When the deciduous pulp is compromised, endodontic treatment should be performed to preserve the integrity and function of the tooth and its supporting tissues.^3^ The instrumentation of the root canal may be performed with manual or mechanical tools.^4^

The instruments that incorporate reciprocating movements were introduced into the market to model the dental channel using only a file. These file instruments have a different mechanism compared with other previously developed files. The system is designed to be used with a reciprocating movement.^5^

Endodontic treatment with rotary systems can contribute significantly to reducing the duration of clinical care for pediatric patients. However, it is essential to evaluate the dentin wear created both by hand and by rotary instruments to analyze the safety of this procedure in different root thirds and in the presence of resorption.^6^ This study aims to demonstrate the potential applications of technological advances in endodontics in pediatric dentistry.

## METHODOLOGY

The sample comprised artificial deciduous teeth with coronary and root pulp (Denarte, São Paulo, SP, Brazil) divided into three groups: group I was instrumented with a manual technique (G1), group II used a roundabout technique (G2), and group III used a reciprocating instrumented technique (G3). The manual instrumentation group used instrumentation techniques such as the scheduling Crown–Down manual, which were performed with the Kerr type files 1st series 21 mm (VDW, Munich, Germany). The instrumentation system used the RECIPROC file Reciproc 25/0.08 (VDW, Munich, Germany), while the instrumentation group's rotary system used the Mtwo files of sizes 10/0.4, 15/0.5, 20/0.6, 25/0.6–21 mm, adapted to the VDW engine (VDW, Munich, Germany).

The procedure to gain access to the root canal was performed by an endodontist, experienced in crown opening with a round bur no. 1014, exploitation of channels C file—Pilot # 10 0.2, 21 mm, to the working length (CT) without resistance, and widening the entrance of the conduits with Gates Glidden drills using no. 2–4. It was then performed at timed instrumentation conduits. We used a stopwatch to measure the instrumentation time in each root canal.

## RESULTS

**Fig. 1:**
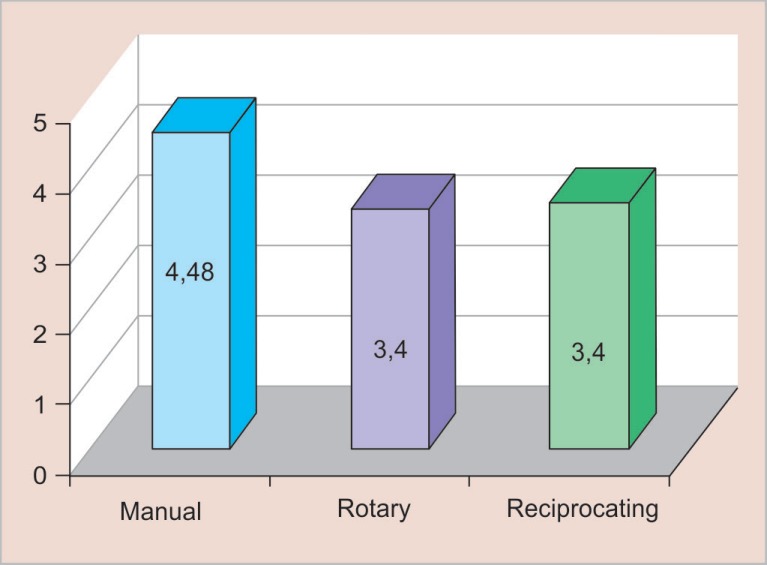
Required time for instrumentation for the three systems

The comparisons between the manual instrumentation group and the rotary and reciprocating systems showed that all the three groups were able to perform the instrumentation of the conduits. The average time required for the manual technique was 4.4 minutes, while with the rotary and reciprocating systems, the average time required was 3.4 minutes (Fig. 1), i.e., the required time for instrumentation of the three systems.

**Fig. 2:**
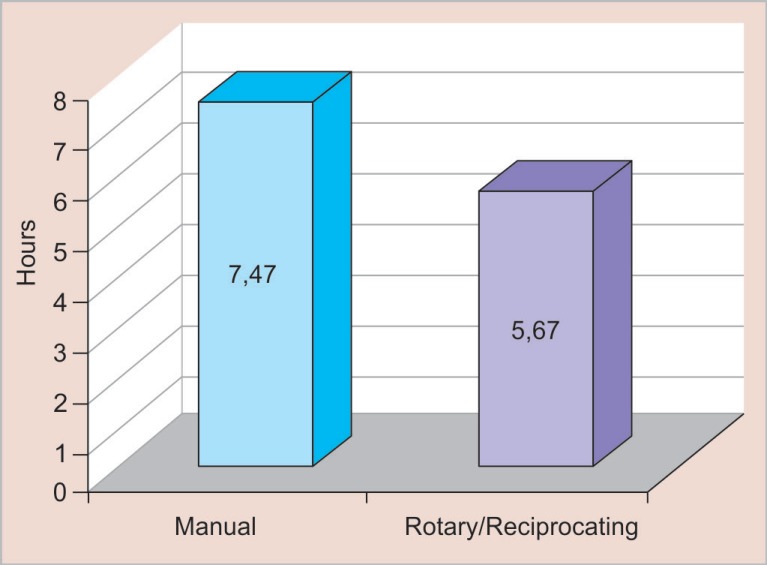
Percentage of time saved between systems (1 month)

## CLINICAL APPLICATIONS

**Fig. 3:**
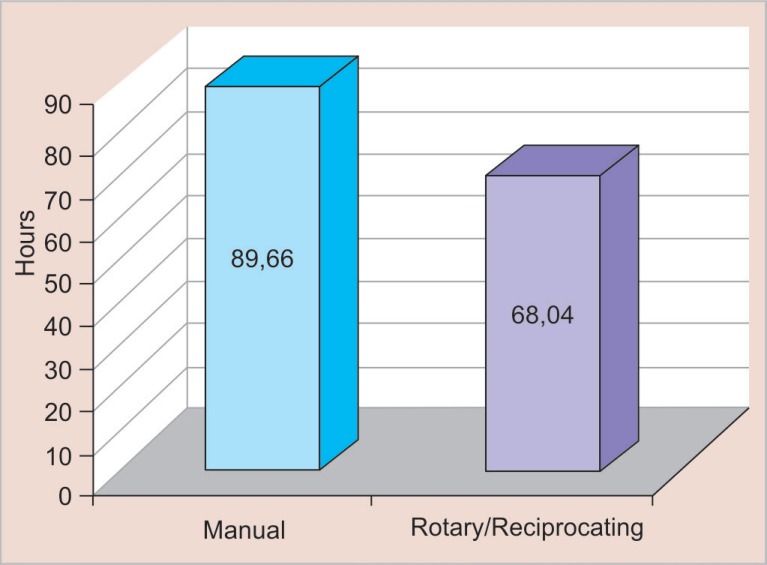
Percentage of time saved between the two systems (one year)

If we consider these procedures implemented in a professional clinical practice for 4 weeks, from the 2nd to the 6th week, and with two patients per day, the rotary and reciprocating systems will have saved approximately 24% of time spent relative to the manual procedure (Fig. 2), the percentage of time saved between systems (1 month). Considering the practice over the course of 1 year, at a rate of two patients per day, the time savings are still approximately 24%. Thus, this economical approach could result in 18 more patients being seen per month, as indicated by the following formula (Fig. 3):

1.8 h = 3,648 s, until 3,648/204 = 17.9–18 patients per month

## DISCUSSION

The main objective of pediatric dentistry's in the pulp treatment is to maintain the integrity and health of dental tissues. This is accomplished through the use of techniques and/or medications that allow the continuation of normal development until exfoliation and respect the particular characteristics of the life cycle of these elements.^7^

Comprehensive endodontic therapy for primary teeth can be challenging due to the peculiar anatomy of these teeth, as well as the child's behavior. For this reason, the length of the root must be correctly determined to minimize the apical periodontitis and possible damage to the permanent successor.^3^

Procedures applied in this study demonstrated that modern endodontic techniques had identical effects in reducing the treatment time. In addition, this can limit the risk of clinical stress as much as possible.

The rotary technique required significantly less time compared to manual instruments.^4,6,8^ This is consistent with observations from other researchers who have noted that the optimization of endodontic treatment in primary teeth is important because it improves treatment quality and decreases clinical time.^3,6,9–13^ The principles of rotary instrumentation for deciduous teeth are the same as for permanent teeth.

The rotary instruments offer several advantages over traditional stainless steel instruments. They are flexible, have more cutting power, provide better maintenance of the original canal shape, considerably reduce deviation tendency or movement of the foramen, and reduce the operative time.^11^

Several studies have been conducted comparing the NiTi instruments’ reciprocating and rotational movements. In evaluating the cyclic fatigue and bending of these instruments, there was a greater resistance observed of files applied in a reciprocal motion relative to the conventional rotational method, longer life-spans for these instruments and a greater ability to maintain the centralized channel. In addition, the instrument's reciprocating movement caused less movement of apical foramen and less extrusion of dentin debris to the periapex.^14–16^ Results are consistent with the study showing that the preparation time with rotary instruments was significantly less than the manual instrumentation, which is a clinically relevant factor for endodontic treatment.

## CONCLUSION

Technological advances have simplified endodontic procedures with regard to the roundabout technique and reciprocation, even in the context of primary dentition, confirming other findings in the literature. However, the good treatment also depends on the reduction or elimination of the infectious agent, appropriate instrumentation, efficient irrigation, and shutter-compatible antibacterial materials, as well as knowledge of the case study. However, well-designed follow-up studies for further statistical investigation are necessary for performing endodontic treatment of primary teeth.

## References

[1] Braga MM,, Oliveira LB, (2009;). Feasibility of the International Caries Detection and Assessment System (ICDAS-II) in epidemiological surveys and comparability with standard World Health Organization criteria.. Caries Res.

[2] Vasconcelos Cunha Bonini GA,, Marcenes W, (2009;). Trends in the prevalence of traumatic dental injuries in Brazilian preschool children.. Dent Traumatol.

[3] Mello-Moura AC,, Moura-Netto C, (2010;). *Ex vivo* performance of five methods for root canal length determination in primary anterior teeth.. Int Endod J.

[4] Silva LA,, Leonardo MR, (2004;). Comparison of rotary and manual instrumentation techniques on cleaning capacity and instrumentation time in deciduous molars.. J Dent Child.

[5] Sasaki EW,, Versiani MA, (2006;). *Ex vivo* analysis of the debris remaining in flattened root canals of vital and nonvital teeth after biomechanical preparation with NiTi rotatory instruments.. Braz Dent J.

[6] Kummer TR,, Calvo MC, (2008;). *Ex vivo* study of manual and rotary instrumen-tation techniques in human primary teeth.. Oral Surg Oral Med Oral Pathol Oral Radiol Endod.

[7] Corrêa FNP,, Corrêa JPNP, (2008;). Tratamento endodôntico em antecessor de dente de Turner.. Rev Inst Ciênc Saúde.

[8] Nagaratina PJ,, Shashikirann D, (2006;). *In vitro* comparison of NiTi rotary instruments in root canal preparations of primary and permanent molar.. J Indian Soc Pedog Prev Dent.

[9] Barr ES,, Kleier DJ, (1999;). Use of nickel-titanium rotary files for root canal preparation in primary teeth.. Pediatr Dent.

[10] Walia HM,, Brantley WA, (1988;). An initial investigation of the bending and torsional properties of Nitinol root canal files.. J Endod.

[11] Ferraz CC,, Gomes NV, (2001;). Apical extrusion of debris and irrigants using two hand and three engine-driven instrumentation techniques.. Int Endod J.

[12] Pettiette MT,, Delano EO, (2001;). Evaluation of success rate of endodontic hand files.. J Endod.

[13] Moura ACVM,, Borelli T, (2013;). Como podemos otimizar a endodontia em dentes decíduos? Relato de caso.. Rev Assoc Paul Cir Dente.

[14] Kuhn WG,, Carnes DL (1997;). Effect of tip design of nickel-titanium and stainless steel files on root canal preparation.. J Endod.

[15] Leonardo MR,, Silva LA, (2008;). *Ex vivo* evaluation of the accuracy of two electronic apex locators during root canal length determination in primary teeth.. Int Endod J.

[16] Kazemi RB,, Stenman E, (1996;). Machining efficiency and wear resistance of nickeltitanium endodontic files.. Oral Surg Oral Med Oral Path Oral Radiol Endod.

